# Bladder Paraganglioma With Rapid Metastasis: A Case Report and Review of the Literature

**DOI:** 10.7759/cureus.100705

**Published:** 2026-01-03

**Authors:** James Turney, Gabrielle R Yankelevich, Matvey Tsivian

**Affiliations:** 1 Department of Urology, Rocky Vista University College of Osteopathic Medicine, Ivins, USA; 2 Department of Urology, Medical University of South Carolina, Charleston, USA

**Keywords:** germline mutation, muscle invasive bladder cancer, paraganglioma, sdhb, sdhb mutation

## Abstract

Bladder paragangliomas (bPGLs) are extremely rare tumors, and we present a unique case of metastatic bPGL that developed within three months. Frequently, these tumors are related to a hereditary syndrome due to germline mutations in succinate dehydrogenase B (SDHB). A unique challenge in this case was related to social determinants of health, which, in this patient, were associated with difficulty maintaining appointments. In treating a patient with hematuria, it is important to consider paraganglioma (PGL) as a differential diagnosis to optimize treatment.

## Introduction

Genitourinary paragangliomas (PGLs) are rare neuroendocrine tumors that arise from sympathetic paraganglia located throughout the genitourinary tract. Bladder paragangliomas (bPGLs) account for over 75% of PGLs found within the genitourinary tract; however, this only represents 6% of all PGLs and less than 0.06% of all bladder malignancies [1]. Clinically, bPGLs often present with a triad of hematuria, hypertension, and episodic headaches, particularly triggered by urination, as a result of catecholamine release [1]. These tumors may arise sporadically or as part of hereditary paraganglioma-pheochromocytoma (PGL-PCC) syndrome. This syndrome is associated with a variety of germline mutations, including succinate dehydrogenase B (SDHB) [2]. Mutations in SDHB are particularly concerning, as they are associated with a higher risk of metastasis and malignant progression, with affected individuals being at greater risk for developing multifocal tumors and extra-adrenal PGLs [2,3]. SDHB encodes a catalytic subunit of the succinate dehydrogenase (SDH) complex, which plays a dual role in the Krebs cycle and mitochondrial electron transport chain. Loss-of-function mutations lead to the accumulation of the oncometabolite succinate, which stabilizes hypoxia-inducible factors (HIFs) and inhibits α-ketoglutarate-dependent dioxygenases, promoting pseudohypoxia, epigenetic reprogramming, and tumorigenesis. This dysregulated cellular environment contributes to the aggressive and metastatic potential observed in SDHB-mutated PGLs [4].

We report a case of metastatic bPGL, which was treated with palliative radical cystectomy. This case highlights the critical importance of genetic testing and follow-up after identification of PGL-PCC, as early detection of germline mutations can influence treatment decisions and long-term surveillance strategies. 

## Case presentation

A 34-year-old African-American female, with no significant family history and a past medical history of asthma, anxiety, and depression, was transferred from a community hospital with hematuria and clot retention, as well as imaging findings of a bladder mass. She reported smoking two to three cigars per day for the past year. The patient had a history of hematuria for over one month and had been prescribed several courses of antibiotics for presumed urinary tract infections (UTIs). She ultimately presented at an outside hospital due to worsening hematuria, a feeling of incomplete emptying, new right flank pain, and worsening dizziness. In the emergency room, she was tachycardic to the 110s, with stable blood pressure. Her labs were notable for leukocytosis of 18, anemia with hemoglobin of 7.5, and a normal creatinine of 0.8. Her abdominal exam was notable for tenderness to palpation in the bilateral lower quadrants, as well as mild right costovertebral angle (CVA) tenderness. 

**Figure 1:**
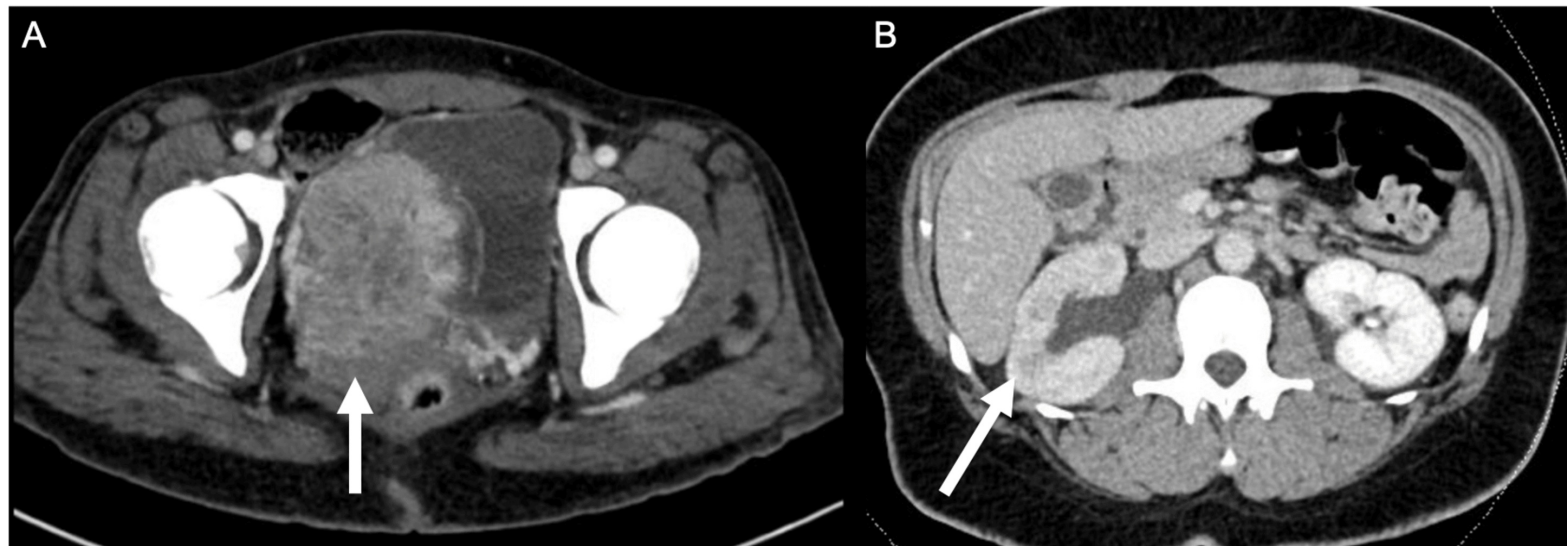
A) Axial view of CT scan showing large volume bladder tumor (white arrow). B) Axial view of CT scan showing right hydronephrosis with delayed nephrogram (white arrow).

Imaging revealed a large bladder mass and right hydronephrosis (Figures 1A-1B). She then underwent a transurethral resection of bladder tumor with clot evacuation. Endoscopically, a very large bladder mass was visualized arising from the right lateral and anterior bladder wall, in close proximity to the ureteral orifice. The operative note reported that the mass did not appear urothelial in nature on gross examination. A right retrograde pyelogram was performed, showing hydronephrosis, and a right ureteral stent was placed. A pelvic exam under anesthesia was unremarkable with a normal appearing vaginal vault and cervix. Pathology from transurethral resection reported a neuroendocrine neoplasm consistent with PGL with positive stains for GATA3, INSM1, S-100, chromogranin A, and SOX-10.

Thereafter, serum catecholamines were obtained, notable for extremely elevated norepinephrine at 23,839 (normal 1,050-4,800), dopamine at 3090 (normal < 240), and free normetanephrine at 15 (normal < 0.9). Urine total metanephrines were obtained, which were also elevated at 13,146 (normal < 1,300).

She underwent a PET-CT DOTATATE-64 scan without evidence of metastatic disease and was referred to endocrinology for preoperative optimization, where she was started on doxazosin and later metoprolol. She had genetic testing and was found to have an SDHB mutation (pathogenic variant in SDHB c.636dupT) consistent with the clinical diagnosis of hereditary PGL-PCC syndrome. 

**Figure 2:**
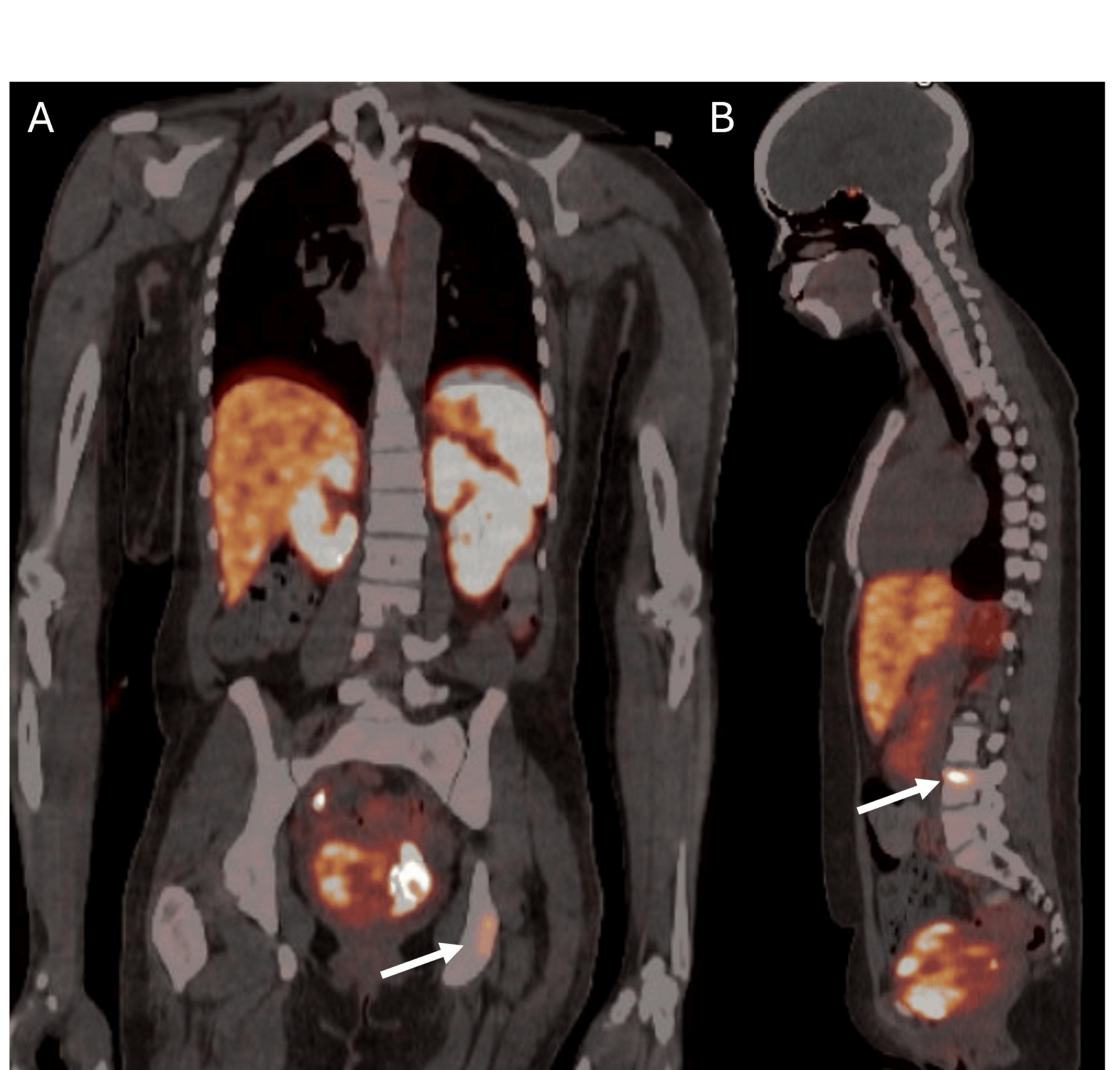
A) Coronal view of PET showing radiotracer avid lesion on L ischium (white arrow). B) Sagittal view of PET showing radiotracer avid lesion on L4 vertebral body (white arrow).

Her planned surgical resection in the form of cystectomy was delayed several times due to her difficulties attending endocrinology appointments for adrenergic blockade titration because of significant social barriers, including lack of insurance coverage, limited transportation, and other barriers. She was lost to follow-up for three months, and when she re-established care, she was admitted to the medicine service for titration of alpha-blockage prior to surgery. During this time, she underwent repeat PET-CT with findings of new radiotracer-avid lesions of the left clavicle, left ischium, and L4 vertebral body (Figures 2A-2B). A multidisciplinary discussion was undertaken, which supported the decision to proceed with surgical resection.

For optimization, she was started on aggressive hydration with IV normal saline and given doxazosin 1 mg twice daily. Per the Endocrine Society, the target goal is a blood pressure of less than 130/80 mmHg when sitting and no less than 80/45 mmHg when standing. The target heart rate is 60-70 beats/minute when sitting and 70-80 beats/minute when standing. Therefore, her orthostatics were monitored, and the doxazosin was up-titrated (patients ultimately can receive up to 16 mg/day). Labetalol 100 mg twice daily was added for tachycardia. 

She was optimized for 10 days prior to surgery, then underwent open radical cystectomy, with sparing of the uterus, ovaries, and vagina. She also underwent pelvic lymph node dissection, ileal conduit urinary diversion, and resection of a pelvic peritoneal nodule. The peritoneal nodule was sent as a frozen section and returned as consistent with metastatic PGL. Final pathology was pT2N0M1 PGL.

The patient's postoperative course was complicated by a postoperative ileus with initial refusal of nasogastric tube (NGT) insertion, but ultimately, an NGT was placed on postoperative day 7 for decompression. A small bowel follow-through (SBFT) was performed, showing contrast in the colon at 24 hours, and ultimately, the NGT was able to be removed on postoperative day 9. The patient remained inpatient for social disposition issues and ultimately was able to be discharged on postoperative day 27. The bilateral ureteral stents and pelvic drain were removed prior to discharge. 

One month postoperatively, the free normetanephrine had declined to almost normal at 1.4, and six months postoperatively, it was within normal limits at 0.68. Unfortunately, to date, the patient continues to experience severe socioeconomic distress, with difficulties including lack of insurance, transportation, and challenges in paying for medications and ostomy supplies. She has been on zoledronic acid (Zometa) infusions for her lytic bone lesions. She is planned for follow-up with medical oncology and for follow-up imaging with PET-CT DOTATATE-64 scan. 

## Discussion

bPGLs are exceptionally rare neuroendocrine tumors, representing less than 0.06% of all bladder tumors [1]. While most reported cases are localized, the presence of metastatic disease, as seen in our patient, is particularly uncommon and often associated with underlying genetic syndromes, such as in our patient [5]. Our case adds to the limited literature on metastatic bPGL and emphasizes the importance of recognizing hereditary syndromes like PGL-PCC early in the diagnostic workup. 

Previous literature has identified a clear association between SDHB mutations and aggressive behavior in bPGLs. In a series of 27 patients with urinary bPGLs, Martucci et al. reported that 14 (52%) harbored germline SDHB mutations [6]. Nearly half of the cohort (48%) developed metastatic disease, including six patients with SDHB mutations, underscoring the malignant potential of this genetic alteration. Similarly, Park et al. analyzed 52 cases and identified nine tumors with SDHB deficiency by immunohistochemistry, six of which were confirmed to carry SDHB mutations by sequencing [7]. These SDHB-deficient tumors exhibited significantly larger size, greater mitotic activity, higher rates of lymphovascular invasion, and a markedly increased incidence of metastasis (22% vs. 2%), compared to SDHB-intact tumors. These findings highlight the critical prognostic implications of SDHB mutations and support the need for early identification, genetic testing, and close long-term surveillance in affected patients. 

While hematuria and hypertensive episodes triggered by micturition are considered classic signs, many patients may present without this full constellation of symptoms, especially in advanced disease [8]. In our patient, the identification of an SDHB mutation was consistent with a diagnosis of hereditary PGL-PCC syndrome, and likely contributed to her tumor’s aggressive behavior. SDHB mutations are well-documented to carry a higher risk for malignant transformation and distant metastasis, compared to other SDHB mutations, with as high as a 40%-60% risk of metastasis [9,10]. The pathophysiology behind the aggressive nature of SDHB-mutated tumors is thought to stem from disruptions in mitochondrial function and accumulation of succinate, leading to pseudohypoxia and activation of angiogenic pathways. This contributes to both tumorigenesis and metastatic potential [9]. 

Surgical resection remains the cornerstone of treatment for bPGLs. In localized tumors, transurethral resection or partial cystectomy may be sufficient, but in cases of extensive or metastatic disease, more radical interventions, such as cystectomy with urinary diversion, may be required [8]. Beilan et al. performed a systematic review of bPGL and found that 20% were treated with transurethral resection, 70% with partial cystectomy, and 10% with radical cystectomy [11]. They were unable to delineate the specific outcomes for endoscopic versus surgical approach, but reported that 14.2% developed a tumor recurrence and 9.4% developed metastatic disease. Overall, they reported that those who presented with localized bPGL had improved survival in comparison to those with locally advanced or metastatic disease on presentation [11]. If transurethral resection is performed, there are no standardized or even proposed guidelines for surveillance reported in the literature. There is even less literature on lymph node dissections, with Purnell et al. reporting only about 40% of surgeries for bPGLs included lymphadenectomy [1]. Regardless, standard pelvic lymph node dissection is recommended [1]. Our decision to proceed with radical cystectomy and ileal conduit was guided by the tumor’s size, invasion, and metastatic potential, particularly given the patient's genetic background [8].

Lastly, it is important to address the social determinants of health that contributed to challenges for this case, including delayed follow-up leading to metastasis. In the general bladder cancer population, Black patients have been shown to receive the lowest percentage of appropriate treatment [12]. Moreover, female gender, black race, unmarried status, unemployed status, and foreign-born status have been independently associated with metastasis at the time of bladder cancer diagnosis [13]. This gender disparity has been shown in the literature, in which women are more likely to have longer delays than men in presentation to a urologist due to presentation with UTI-like symptoms [14-16]. Many of these determinants were present in our case and contributed to delays in care.

This report is limited by its single-patient nature and lack of long-term follow-up at this time. Future research is needed to better characterize the long-term outcomes and recurrence rates following radical cystectomy for metastatic bPGL, particularly in SDHB mutation carriers. Additionally, collaborative efforts to compile multi-center case series may help guide standardized approaches to the management and surveillance of this rare disease entity. 

## Conclusions

This case underscores several key clinical lessons. First, guidelines recommend genetic testing for all patients diagnosed with PGL, given the high prevalence of hereditary syndromes associated with these tumors. Second, early identification of SDHB mutations can inform both treatment aggressiveness and long-term surveillance for multifocal or recurrent disease. Lastly, the need for multidisciplinary care, including urology, oncology, endocrinology, and genetics, is essential in optimizing outcomes in patients with hereditary PGL-PCC syndromes.
